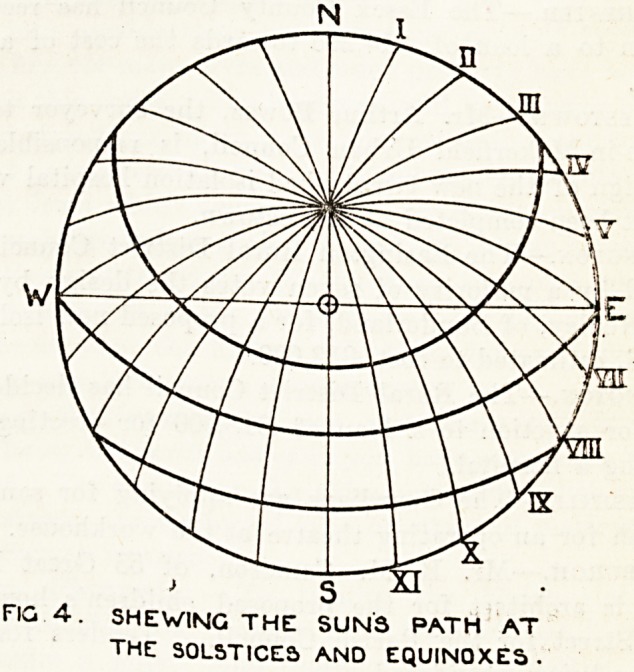# Sun-Planning for Hospitals

**Published:** 1912-11-02

**Authors:** 


### HOSPITAL ARCHITECTURE AND CONSTRUCTION.

**[Communications on this subject should be marked "Architecture" in the left-hand top corner of the envelope.]**

### Sun-planning for Hospitals.

IT is of fundamental importance in designing all buildings, and particularly those forming the hospital pavilions, to study on right lines the subject of sunlight and shadows cast thereby. What is the correct aspect for a ward in this country and why it is correct we shall try to explain and demonstrate

FIG. 1. APPARENT PATH OF THE SUN AT SUMMER SOLSTICE.

in this article. Some reader may say that certain aspects are acknowledged to be correct, but will be at a loss to explain why others have in important buildings been favoured. For instance, Dr. Knauff gives a very exhaustive account * of the arguments which ultimately led to placing the pavilion axes at Heidelberg University Hospital as nearly east and west as the configuration of the site would

FIG. 2. APPARENT PATH OF THE SUN AT THE EQUINOXES.

permit; the Friedrichshain authorities, after mature deliberation, arrived at an exactly opposite conclusion, and orientated the wards north and south. Further, in a list of some thirty-eight hospitals given by Mouatt and Snell thirteen are described as being placed north and south, fifteen are placed approximately east and west, six approximately north-west and south-east, and the four remaining examples north-east and south-west. These instances go to prove that there is considerable difference of opinion as to what is the correct alignment of the pavilion

FIG. 3. APPARENT PATH OF THE SUN AT THE WINTER SOLSTICE.

unit, but the curative and healthful effect of the sun's rays is acknowledged by all.

Fig. 1 illustrates in diagram the apparent course of the sun at the summer solstice (June 21), fig. 2 the same at the vernal and autumnal equinoxes (March 21 and September 22), and fig. 3 at the winter solstice (December 21).

The natural conclusion from a study of these

FIG 4. SHEWING THE SUNS PATH AT THE SOLSTICES AND EQUINOXES.

diagrams is that the pavilion's long axis should be true north and south, but another factor here develops, and that is that the path of the sun is not always in a straight course from sunrise to sunset; in the summer solstice it traces a hollow cone, at the equinoxes a plane, and at the winter solstice a convex cone; this point is more clearly demonstrated in fig. 4, which illustrates the path

* *Das Neue Academische Krankenhaus in Heidelberg.* München. 1879.

of the sun in a diagram of the celestial sphere. The ward aligned north and south receives least sunlight in winter, when it is most desirable, and the maximum in summer, when it is least wanted. In the east and west alignment the results are reversed, showing a maximum in winter and a minimum in summer on one side only; therefore, if we judge only on the basis of amount of sunlight received by windows, undoubtedly the best position is, as concluded by Dr. Knauff, due east and west; but there is the disadvantage that this position involves an area of complete shadow on one side of the pavilion during one-half of the year, and, further, that a greater distance is necessary between the pavilions than in any other orientation.

The north and south position has as many disadvantages as the east and west alignment, and nothing gained; the windows admit but little sunlight in winter and too much in summer, so that if this position is ever adopted the wards should invariably have windows at the south end to entrap the winter sun.

There remain yet to be considered the two intermediate positions—viz., north-east south-west and north-west south-east. In these there is but little variation in the amount of sunlight registered, and by the addition of south-east or south-west windows the amount of winter sun obtained is satisfactory. Also, in either of these positions it would be possible to place the pavilions closer together and yet have three walls flushed by sunlight at some hour of the day throughout the year. The prevailing winds in this country being from the south-west would favour the ward having its long axis north-east and south-west, so that as little area as possible should be directly exposed to the prevailing winds, while the flank walls would be well flushed with moving air.

## Figures and Tables

**Fig. 1. f1:**
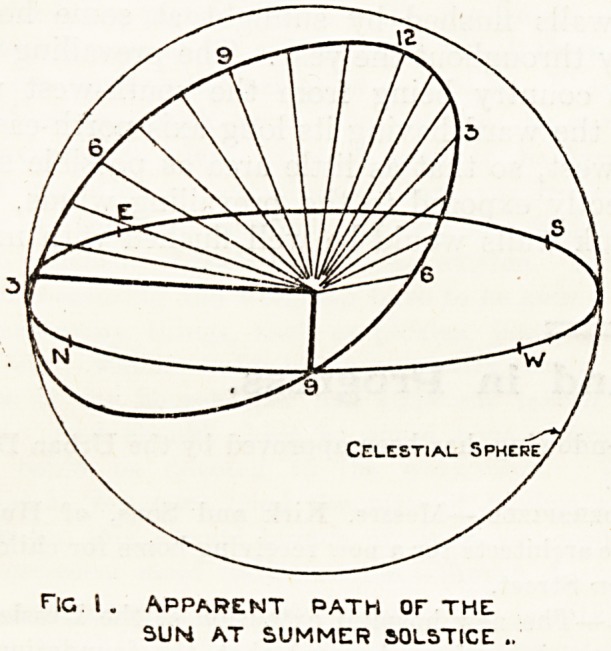


**Fig. 2. f2:**
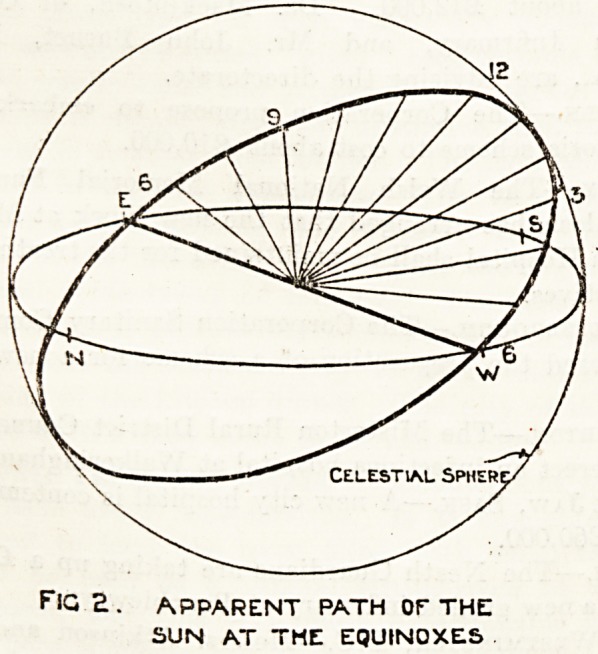


**Fig. 3. f3:**
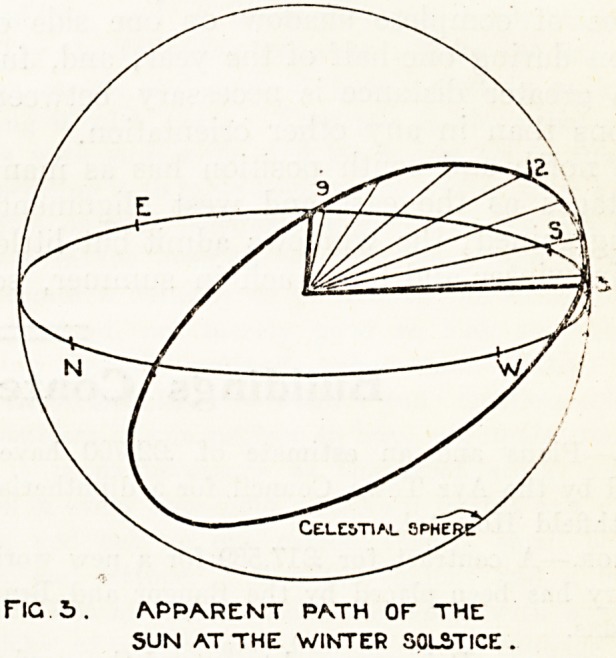


**Fig. 4. f4:**